# Identification of an IGF2BP2-Targeted Peptide for Near-Infrared Imaging of Esophageal Squamous Cell Carcinoma

**DOI:** 10.3390/molecules27217609

**Published:** 2022-11-06

**Authors:** Wenbin Shu, Yitai Xiao, Lizhu Wang, Mingzhu Liang, Zhihong Li, Xiangwen Wu, Qingdong Cao

**Affiliations:** 1 Department of Cardiothoracic Surgery, The Fifth Affiliated Hospital of Sun Yat-Sen University, Zhuhai 519000, China; 2 Guangdong Provincial Key Laboratory of Biomedical Imaging and Guangdong Provincial Engineering Research Center of Molecular Imaging, The Fifth Affiliated Hospital of Sun Yat-Sen University, Zhuhai 519000, China; 3 Department of Radiology, The Fifth Affiliated Hospital of Sun Yat-Sen University, Zhuhai 519000, China; 4 School of Medicine, South China University of Technology, Guangzhou 510006, China

**Keywords:** IGF2BP2, esophageal squamous cell carcinoma, peptide, NIR imaging

## Abstract

Esophageal squamous cell carcinoma (ESCC) is one of the most lethal malignancies globally. Peptide-based tumor-targeted imaging is critical for ESCC imaging. In this study, we aim to identify a peptide-targeting IGF2BP2 that specifically binds to human ESCC for near-infrared imaging of esophageal cancer. Applying phage display techniques, we identified a peptide target for IGF2BP2 which was confirmed to be highly expressed in ESCC cell lines or tumor tissue and may serve as an imaging target for ESCC. We conjugated the peptide to the NIRF group, Cy5, and further evaluated the targeting efficacy of the probe at a cellular level and in animal tumor models. The Cy5 conjugated peptide (P12-Cy5) showed a high binding affinity to human ESCC cells in vitro. In vivo, optical imaging also validated the tumor-targeting ability of P12-Cy5 in KYSE-30-bearing subcutaneous ESCC tumor models. Furthermore, the results of biodistribution showed a significantly higher fluorescence intensity in tumors compared to scrambled peptide, which is consistent with in vivo observations. In summary, an IGF2BP2-targeted peptide was successfully identified. In vitro and in vivo experiments confirmed that P12-Cy5 has high affinity, specificity and tumor-targeting properties. Thus, P12-Cy5 is a prospective NIR probe for the imaging of ESCC.

## 1. Introduction

Esophageal cancer (EC) is one of the most lethal malignancies, ranking sixth in cancer mortality rates globally [1]. In Asian countries, esophageal squamous cell carcinoma (ESCC) is the predominant pathological type. Multimodal treatments, based on extensive esophagectomy and lymphadenectomy, have remarkably improved survival rates [2]. Early ESCC presents with insidious symptoms, and most patients are not diagnosed until the late stages, which limits the options for radical treatment [3,4]. Despite the low incidence, the survival rate of ESCC is extremely low, which highlights the primary importance of the early treatment of ESCC. Novel imaging systems have emerged in response to the limited effectiveness of conventional white light endoscopy in the routine diagnoses of ESCC. Due to the remarkably low absorption and autofluorescence of biological tissues within the near-infrared (NIR) spectrum (650–900 nm), near-infrared fluorescence (NIRF) imaging has been employed to visualize various biomolecules which are closely related to the disease’s processes at the cellular and even molecular levels [5,6].

A concept that has recently emerged is the exploitation of specific biochemical molecules altered during cancer progression to develop novel molecular imaging methods. The availability of some biomarkers for ESCC may lead to the emergence of new surveillance methods for identification and detection in high-risk populations. As an m6A-binding protein that enhances mRNA stability in an m6A-dependent manner, human insulin-like growth factor 2 (IGF2) mRNA binding protein 2 (IGF2BP2) is involved in the initiation and development of several malignant cancers [7,8]. Functioning as a tumor promoter, IGF2BP2 is significantly upregulated in EC tissues and is considered to be associated with poor prognosis [7,9]. In light of the high expression rate, molecular tracers that target IGF2BP2 may contribute to the precise imaging of ESCC.

As the key factor in NIR fluorescence imaging, NIR imaging probes usually consist of NIR dyes and targeting moieties (including antibodies and their fragments [10], peptides [11], small molecules [12], etc.) that interact specifically with molecules during tumorigenesis and evolution. Peptides stand out among many targeted molecules due to their lower immunogenicity, greater tissue penetration, faster blood clearance, and relatively simple production methods [13,14]. Incorporating the antigen-recognizing ability of recombinant proteins, phage display technology is an efficient screening system for the generation of peptides or antibodies against specific molecules or tumor structures; thus, it holds great promise in the field of monoclonal antibody and tumor-specific peptide development [15,16].

In this study, we identified a 12-amino-acid peptide targeting IGF2BP2 which specifically binds to human ESCC. We conjugated the peptide to an NIRF group Cy5 and further evaluated the targeting efficacy of the probe at the cellular level and in animal tumor models.

## 2. Results

### 2.1. IGF2BP2 Expression in Cancer Cell Lines and Tumor Tissue

**Figure 1:**
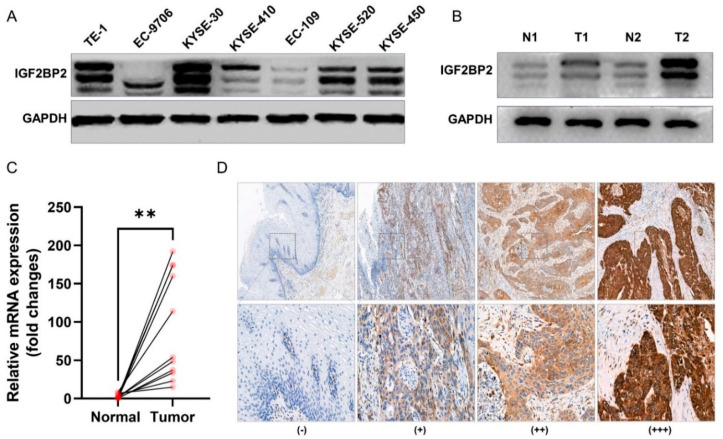
IGF2BP2 expression in ESCC cancer cell lines and tumor tissue. (**A**) Western blotting of IGF2BP2 in esophageal cancer cell lines. (**B**) Western blotting of IGF2BP2 in esophageal cancer tumor tissues (T) and normal tissues (N). (**C**) *IGFBP2* mRNA expression in tumor tissues and normal tissues from 11 patients. ** *p* < 0.01. (**D**) Representative IHC staining images of IGF2BP2 expression in ESCC tissues. −: negative staining, +: weak positive, ++: medium positive, +++: strong positive. Magnification, up 50×, down 200×.

Western blotting was performed to quantify the expression of IGF2BP2 in ESCC cell lines and tumor tissue. As indicated in Figure 1A, the esophageal cancer cell lines exhibited varying degrees of IGF2BP2 overexpression, with KYSE-30 expression being the highest among all the displayed esophageal cancer cells. Western blotting of the patient-derived tumor tissue also showed that IGF2BP2 expression was relatively higher in tumor tissue than in paracancerous tissue (Figure 1B). Retrieval results from the GEPIA database (http://gepia.cancer-pku.cn/, accessed on 11 May 2022.), a comprehensive website for gene expression profiling, revealed that the expression of *IGF2BP2* is significantly elevated in ESCC compared to normal tissue (Figure S1), which was also verified by the results of mRNA expression in tumors and paracancer tissues derived from 11 ESCC patients (Figure 1C). To investigate the positive rate of IGF2BP2 in ESCC, we also examined the positive rate of IGF2BP2 in 98 patients with ESCC by IHC. The results showed that whilst the positive rate of IGF2BP2 in ESCC tissue reached 96.9% (95/98) (Figure 1D), the IGF2BP2 expression was not correlated with clinicopathological parameters, such as age, gender, tumor-node-metastasis (TNM) stage and differentiation (Table 1). All these results suggested that IGF2BP2 is an important tumor marker that is highly expressed in ESCC in either cells or tumor tissue, implying that it may be a potential target for ESCC imaging.

### 2.2. Identification of IGF2BP2-Targeted Peptides

**Figure 2:**
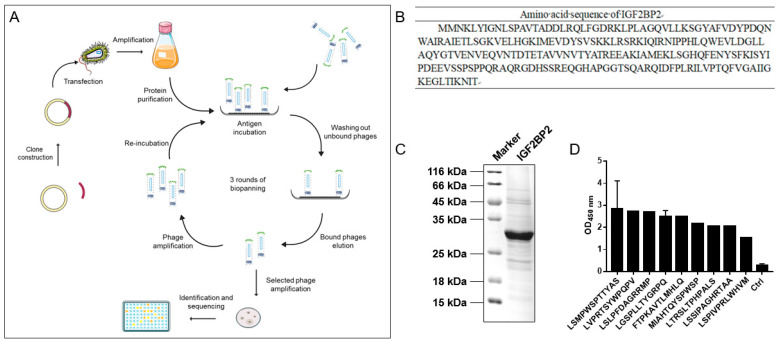
Identification of IGF2BP2-targeted peptides. (**A**) A flow chart for screening of IGF2BP2-targeted peptides. (**B**) Amino acid sequence of IGF2BP2 protein. (**C**) Coomassie brilliant blue staining of IGF2BP2 protein after expression and purification. (**D**) The binding affinities of the nine selected phages. The OD values were analyzed by phage ELISA. Standard deviations appeared in some columns because some repeated sequences appeared several times.

Figure 2A concisely illustrates the procedure of peptide screening. The amino acid sequence of the IGF2BP2 protein was obtained according to the gene sequence of IGF2BP2 (Figure 2B). The IGF2BP2 protein was successfully purified, producing a consistent and predicted molecular weight of approximately 29 kDa, illustrated by the Coomassie blue staining (Figure 2C). A total of three rounds of incubations and screenings were carried out. Significant enrichment of recovered phages was observed. After the final round of screening, 19 clones were randomly selected and validated by ELISA, and high-throughput sequencing technology was performed to sequence the phage. Two highly repeated peptide sequences were identified displaying high absorbance intensity at 450-nm wavelengths, which is significantly higher than those produced by the control (Figure 2D). The higher frequency of the peptide sequence indicated the effective enrichment during the screening process. The peptide sequence LSMPWSPTTYAS (dubbed as P12), with an OD value equivalent to 10 times that of the control, was selected as the best candidate for further study, whereas a peptide with a scrambled sequence (dubbed as C12) was used as the control peptide.

### 2.3. Synthesis of IGF2BP2-Targeted Peptide

**Figure 3:**
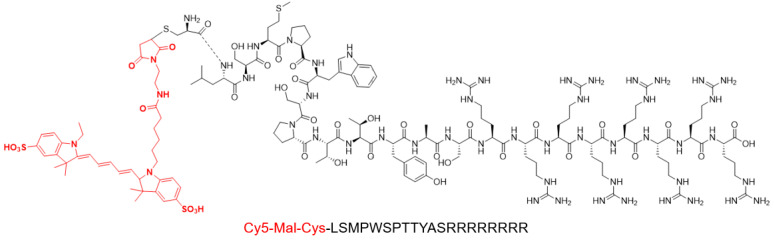
Chemical structure of P12-Cy5. The screened P12 peptide was conjugated to an arginine-penetrating peptide (RRRRRRRR) at the C-terminus and a Cy5 fluorescent group (marked in red) at the N-terminus.

As illustrated in Figure 3 for the P12-Cy5 chemical molecular structure, the screened P12 peptide was conjugated to an arginine-penetrating peptide (RRRRRRRR) at the C-terminus and a Cy5 fluorescent group (marked in red) at the N-terminus. The mass-to-charge ratio (m/z) of P12-Cy5 was confirmed by mass spectrometry as 3472.03 (Figure S1A). The peptide was purified by HPLC with a retention time of 11.53 min (Figure S1B).

### 2.4. Binding of IGF2BP2-Targeted Peptide to Cancer Cell Lines

**Figure 4:**
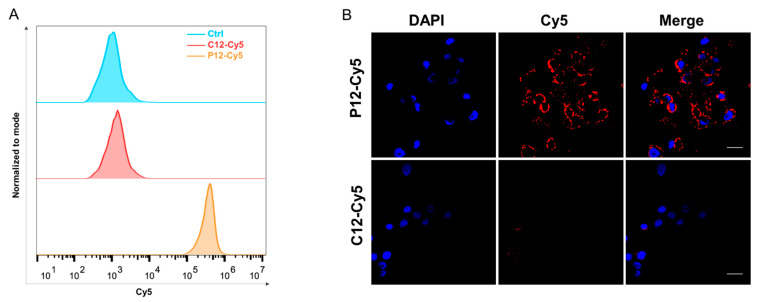
Binding of IGF2BP2-targeted peptide to cancer cell lines. (**A**) Flow cytometric analysis of KYSE-30 cells after treatment with C12-Cy5 or P12-Cy5 peptide. (**B**) Confocal images of KYSE-30 cells after incubation with C12-Cy5 or P12-Cy5 peptide. Scale bar: 20 μm.

The in vitro specificity of the peptides was validated by flow cytometry and immunofluorescence staining. As indicated in Figure 4A, flow cytometric analysis shows a peak shift in P12-Cy5-treated KYSE-30 cells, rather than in C12-Cy5-treated KYSE-30 cells. Consistent with flow cytometry, immunocytochemistry also shows a strong binding in the cytoplasm of IGF2BP2-positive KYSE-30 cells. In contrast, almost no fluorescence was observed in C12-Cy5-treated KYSE-30 cells (Figure 4B). These data indicated that IGF2BP2-targeted peptide P12-Cy5 binds to IGF2BP2-positive cells.

### 2.5. NIRF Imaging in KYSE-30 Bearing Xenograft Models

**Figure 5:**
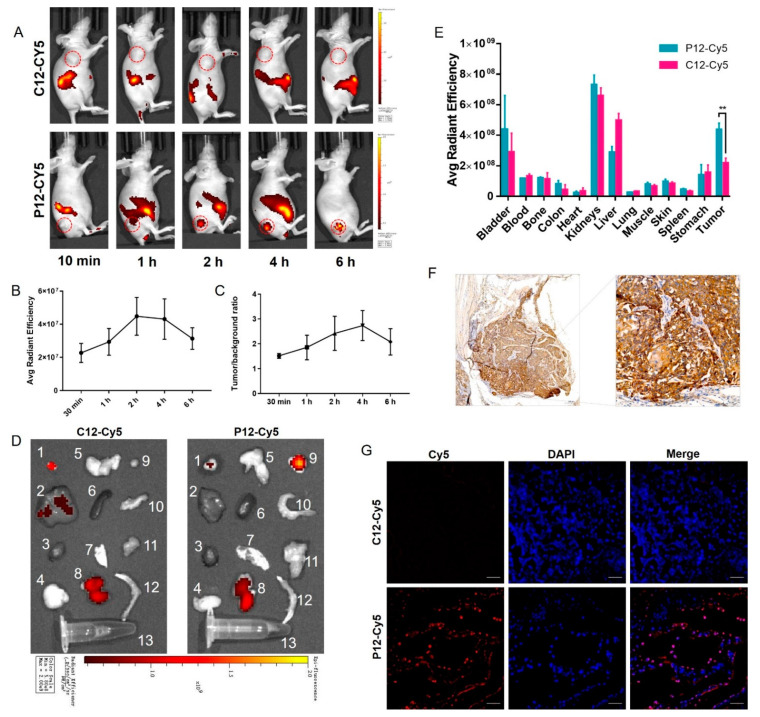
NIRF imaging in KYSE-30 bearing xenograft models. (**A**) Representative NIRF images of KYSE-30 tumor-bearing mice (n = 3) postinjection with P12-Cy5 and C12-Cy5 at different time points. (**B**,**C**) The MFI and TBR quantified from ROI in P12-Cy5 administrated mice (n = 3). (**D**) NIRF images of resected tumors and main organs 2 h postinjection of P12-Cy5 and C12-Cy5 (n = 3). Serial numbers indicate different organs and tissues. 1: bladder, 2: liver, 3: heart, 4: lung, 5: stomach, 6: spleen, 7: skin, 8: kidneys, 9: tumor, 10: colon, 11: muscle, 12: bone, 13: blood. (**E**) Quantitative biodistribution of P12-Cy5 and C12-Cy5 quantified from resected tumors and main organs (n = 3). (**F**) Representative images of IHC staining in tumors resected after imaging. Magnification, left 50×, right 200×. (**G**) Immunofluorescence images of the frozen section from KYSE-30 tumor tissues after imaging. Scale bar: 100 μm.

NIRF imaging was performed in KYSE-30 bearing esophageal cancer xenograft models by intravenous injection of Cy5-peptide in vivo. In comparison to C12-Cy5, P12-Cy5 exhibited a significantly higher tumor accumulation in KYSE-30 xenografts at different time points postinjection, with tumor contrast gradually increasing over time (Figure 5A). Quantitative analysis showed that the mean fluorescence intensity (MFI) of P12-Cy5 peaked after 2 h, with TBRs gradually increasing over time (Figure 5B, C). Ex vivo optical imaging of the tumors and main organs was performed 2 h postinjection. The quantification of the region of interest (ROI) corroborated the visualization of in vivo optical imaging (Figure 5D). Biodistribution indicated that P12-Cy5 showed prominent renal clearance. The enrichment of P12-Cy5 in the KYSE-30 tumors was high in all organs except the urinary organs (Figure 5E). Consistent with the results of Western blotting, the IHC of excised tumors after imaging also demonstrated high expression of IGF2BP2 in tumor tissues (Figure 5E). Immunofluorescence also showed the strong binding of P12-Cy5 to IGF2BP2-positive tumors (Figure 5G), which further corroborated the imaging results. These results suggested that P12-Cy5 has excellent IGF2BP2-positive tumor-targeting potential.

## 3. Discussion

With the advances in precision medicine and high-tech imaging instruments, there is a general consensus that the specific monitoring of key molecules, which reflect tumor information at the cellular or subcellular levels, is superior to conventional imaging techniques that reflect anatomic structural changes [17]. The advent of molecular imaging has provided additional opportunities for non-invasive observation of aberrant molecular events in vivo. As an important component of the tumor targeted probe, targeting molecules (including antibodies and their fragments [10], peptides [11], and small molecules [12]) play the pivotal role of molecular probe. Due to their smaller molecular weight, peptides appear to bind to more hidden antigenic epitopes that other macromolecules cannot reach [13]. Novel peptide-based molecular probes have facilitated cancer detection immensely, owing to the advent and rapid evolution of phage display technology [18,19]. Arg-Gly-Asp (RGD) peptide analogs, which target integrin α_Ⅴ_β_3_, have shown tremendous potential in the preliminary clinical exploration of the characterization of tumor angiogenesis [20,21] and have thus encouraged the exploration of a series of tumor-targeting peptides.

Applying phage display techniques, we identified a peptide target for IGF2BP2, which was confirmed to be highly expressed in ESCC cell lines or tumor tissue and may serve as an imaging target for ESCC. The consideration of generalizability cannot be ignored for studies at the cellular level. We chose IGF2BP2, a high-expression ESCC cell line, for cellular and in vivo experiments, which is desirable but not necessarily universal to all ESCC patients. To reveal the expression of IGF2BP2 in clinical patients, we verified the expression of IGF2BP2 in 98 ESCC tissues and showed that the positive rate of IGF2BP2 in ESCC was as high as 96.9%, which revealed that IGF2BP2 may be an ideal target for ESCC imaging. The Cy5 conjugated peptide (P12-Cy5) showed a high binding affinity to human ESCC cells in vitro. In vivo, optical imaging also validated the tumor-targeting ability of P12-Cy5 in KYSE-30-bearing subcutaneous ESCC tumor models. Within a few hours, P12-Cy5 showed rapid tumor-targeting ability and high-contrast imaging that lasted for 24 h. Furthermore, the results of biodistribution showed significantly higher fluorescence intensity in tumors compared to a scrambled peptide, which is consistent with in vivo observations. Notably, the uptake of P12-Cy5 was relatively higher in the kidney than in other organs, including the tumors, which may be attributed to the fact that the kidney may be the primary excretion pathway. This is consistent with what has been reported in a series of other publications [22,23,24,25].

With the development of molecular biology, the diagnosis and treatment of tumors has been transformed from the traditional macroscopic level to another battlefield tailored to tumor microscopic molecules, which undoubtedly provides tremendous prospects for our screened peptide to make a grand entrance in ESCC diagnosis and treatment. The property of peptides to easily couple with other chelating groups allows them to be readily designed as agents for other imaging modalities, such as positron emission computed tomography (PET) imaging [24] or magnetic resonance imaging (MRI) [26]. Considering that IGF2BP2 is expressed in the cytoplasm, we conjugated an arginine-penetrating amino acid sequence to the peptide, which will endow the peptide with the potential to reach the interior of the cell. The high positive rate of IGF2BP2 in ESCC also makes future use of chemotherapeutic agents or radionuclides coupled to the peptide for ESCC therapy an attractive prospect, which has been stated in earlier studies [27].

A remaining point worth mentioning pertains to the role of IGF2BP2 in ESCC. N6 methyladenosine (m6A) RNA methylation regulators have been reported to serve an essential role in the development of tumors, yet their function in EC has not been fully elucidated. With the development of high-throughput sequencing, sporadic studies have revealed that m6A regulators METTL3 [28], FTO [29], and HNRNPA2B1 [30] are aberrantly expressed in EC and affect the metastasis and invasion of a tumor by affecting the stability of mRNA. In our study, we found that the positive rate of IGF2BP2 in esophageal cancer tissue was up to 96.9%, implying that IGF2BP2 may be somehow involved in ESCC tumorigenesis. Unfortunately, prognostic data were not available for this group of patients, so it is not clear whether IGF2BP2 affects the prognosis of ESCC. Further research is required to fill the gap in this area.

Additional issues need to be addressed in our study. Due to the unproven clinical safety of the near-infrared fluorescent dye Cy5, the clinical application of this dye remains concerning. Approved by the Food and Drug Administration (FDA) for clinical practice [31], indocyanine green (ICG) may be considered a preferable alternative. Given the better tissue penetration and tumor signal-to-noise ratio, NIR in the second window (NIR-II) tumor-targeted imaging obviously holds greater potential for clinical translation [32,33]. Imaging of deep tumors with NIR-II dyes such as IRDye800cw will undoubtedly become more widespread [5,10]. In addition, short peptides have demonstrated their superior performance in tumor diagnostic applications [11,24]. However, the binding affinity of peptides is not on par with that of specific antibodies so far. The lack of direct data on the affinity between the P12 peptide and the IGF2BP2 antigen prevented us from quantitatively comparing the affinity of the P12 peptide with commercially available monoclonal antibodies. Further studies on applying enzyme-linked immunosorbent assay (ELISA) or surface plasmon resonance (SPR) might tackle these shortcomings. Thirdly, the tumor tissues and cell lines we obtained were all esophageal squamous carcinoma, and expression of IGF2BP2 on esophageal adenocarcinoma was not performed in our study, although some studies have demonstrated that IGF2BP2 is also highly expressed in adenocarcinoma [34]. Finally, it is well known that most patients with EC are already in the middle or late stages of the disease at the time of diagnosis [4,35]. Although IGF2BP2 has been shown in the literature to be highly expressed in high-grade dysplasia (HGD) or Barrett’s esophagus, which are considered to be precancerous lesions of EC [34,35], it is unknown whether the targeted peptide can be used for the diagnosis of precancerous lesions. This is one of the main focuses of our subsequent research.

## 4. Materials and Methods

### 4.1. Cells and Mice

Human esophageal cancer cell lines EC-9706, EC-109, and TE-1 were acquired from the Institute of Basic Medical Science, Chinese Academy of Medical Sciences, whereas KYSE-30, KYSE-410, KYSE-450, and KYSE-520 were obtained from the DSMZ (Deutsche Sammlung von Mikroorganismen und Zellkulturen). All cells were maintained in an incubator containing a humid atmosphere of 5% CO_2_ at 37 °C. All cells were cultured in RPMI-1640 medium supplemented with 10% fetal bovine serum (FBS) and penicillin-streptomycin solution. Mice aged 4 weeks were purchased from Guangdong Medical Laboratory Animal Center (Guangdong, China). Subcutaneous tumor models were constructed by subcutaneously injection of about 1 × 107 cells with a 20% suspension of Matrigel (Corning, NY, USA). All animal experiments were performed under a protocol approved by the Animal Ethics Committee of The Fifth Affiliated Hospital of Sun Yat-sen University.

### 4.2. Peptide Screening

For the construction of the IGF2BP2 protein, the IGF2BP2 gene was obtained from the NCBI Gene database (https://www.ncbi.nlm.nih.gov/, accessed on 22 June 2021) and cloned into the pET28-a vector (+) between the BamHI and EcoRI sites and transformed into the BL21(DE3) competent *E. coli* cells. The selected clone was cultured in a Luria broth (LB) medium containing ampicillin at 37 °C until reaching an OD value of 0.6–0.8, followed by adding 1 μM IPTG into the culture to induce expression. The bacterial crude culture was harvested and centrifugated for 10 min at 5000 rpm, and the precipitate was subsequently resuspended with a lysis buffer containing 8 M urea and 50 mM Tris (pH 7.4). After complete decomposition under high pressure, the lysed bacterial was then centrifuged at 15,000 rpm for 30 min and loaded onto a Ni-resin binding (affinity chromatography) column (GE Healthcare). The recombinant IGF2BP2 protein was eluted with a high stringent buffer containing 300 mM imidazole and examined by 10% SDS-PAGE and Coomassie brilliant blue staining. The purified IGF2BP2 protein was coated in a 96-well plate for subsequent peptide screenings.

A phage display library (Ph.D.™-7 Phage Display Peptide Library Kit, E8100S, New England Biolabs, MA, USA) was employed, as previously described, to select and identify peptides that bound to the IGF2BP2 protein. In brief, the purified IGF2BP2 protein was coated onto ELISA plates, and a phage peptide library kit was applied for three rounds of screening. Bound phages were eluted and precipitated by polyethylene glycol (PEG)/NaCl, followed by amplification in E. coli as previously described. After three rounds of screening, the target clones were selected for DNA sequencing to obtain the final target amino acid sequence. The peptide sequence with the highest OD value was selected to link the arginine-penetrating peptide (RRRRRRRR) at the C-terminus and Cy5 fluorescent group at the N-terminus. All peptides were synthesized by Apeptide Co.,Ltd. (Shanghai, China) using solid-phase Fmoc chemistry and purified using high-performance liquid chromatography (HPLC) and electrospray ionization mass spectrometry at a minimum purity of 95%.

### 4.3. SDS-PAGE and Western Blot

As for cells and tumor tissue, protein was extracted using RIPA lysis buffer (P0013E, Beyotime, Shanghai, China) on ice. The protein concentrations were determined by the BCA Protein Assay Kit (P0012, Beyotime, Shanghai, China) according to the manufacturer’s instructions. After boiling for 10 min at 98 °C, protein samples with loading buffer were loaded for electrophoresis with an appropriate voltage for 3 h.

The gels were transferred onto polyvinylidene difluoride membranes (Solarbio, Beijing, China) at 300 mA for complete protein transfer. The membranes were blocked with 5% skim milk and then incubated with primary antibodies at 4 °C overnight. After being rinsed 3 times with PBST, the membranes were incubated with horseradish peroxidase (HRP)-conjugated anti-rabbit/mouse IgG (Zhongshanjinqiao, Beijing, China) for 1 h at room temperature. The immunoreactive bands were visualized by the ECL chemiluminescence system (Bio-Rad, CA, USA) after spreading SuperSignal West Pico Chemiluminescent Substrate (Thermo Fisher Scientific, MA, USA) for a few seconds.

### 4.4. Flow Cytometry

When the cells in the 6 cm dish were 80–90% grown, they were harvested with Accutase (Sigma-Aldrich, MO, USA) and incubated with Cy5 conjugated peptides at a concentration of 50 μM for 15 min at room temperature, protected from light. After incubation, the cells were washed 3 times with ice-cold PBS and resuspended in 200 µL of PBS. Flow cytometry was performed on a CytoFLEX LX flow cytometer (Beckman Coulter, IN, USA) and eventually, 10,000 gated cells were collected for analysis. The results were finally analyzed by flow cytometry using Flow Jo software (v7.6, BD Biosciences, OR, USA).

### 4.5. Immunofluorescence Staining

Approximately 5 × 104 cells were seeded onto fibronectin-coated coverslips in 24-well plates and fixed with 4% paraformaldehyde until the cells reached about 50% confluence. After permeabilization with 0.5% Triton X-100 (Millipore, MA, USA) for 10 min, the cells were blocked with 5% BSA for 0.5 h and subsequently incubated with Cy5 conjugated peptides at a concentration of 80 μM overnight at 4 °C. The next day, 1 μg/mL of diaminophenyl indole (DAPI) was added to stain the nucleus after washing with phosphate-buffered saline (PBS). Thereafter, the coverslips were mounted with ProLong Gold anti-fade (Invitrogen P26930, CA, USA), and the visualizations were conducted using confocal laser-scanning microscopy (Zeiss LSM880, Oberkochen, Germany).

### 4.6. Immunohistochemistry

Samples were fixed with formalin and embedded with paraffin, then cut into 5 μm sections. The sections were deparaffinized in xylene and hydrated in a gradient concentration of ethanol and distilled water. Antigen retrieval was carried out under high pressure with a boiling citrate buffer (pH 6.0, MaxVision, Fujian, China). After being blocked with 5% goat serum for 30 min, all samples were incubated with anti-IGF2BP2 antibodies (11601-1-AP, Proteintech, IL, USA) at 4 °C overnight. Subsequently, the sections were washed 3 times with PBST and incubated with HRP-conjugated rabbit anti-rabbit antibodies (MaxVision, Fujian, China) at room temperature for half an hour. The chromogenic reaction was performed by diaminobenzidine (DAB) (Maxion, Fujian, China), followed by staining with hematoxylin (Zhongshanjinqiao, Beijing, China). The visualizations were conducted using a slide scanner system (3DHistech Ltd., Budapest, Hungary) and images were captured and analyzed by Pannoramic Viewer (3DHistech Ltd., Budapest, Hungary). The histochemistry score (h-score), automatically quantified by the software, was applied for assessing the intensity of the staining, according to which the expression was classified as negative (h-score < 50), weak (50–99), medium (100–199), or positive (200–300) [10].

### 4.7. Near-Infrared Fluorescence Imaging

Mice bearing subcutaneous esophageal tumors were injected with an equivalent of 50 μg Cy5-peptide via a tail vein. After circulation, the mice were anesthetized with isoflurane, and fluorescence images were captured at different time points of 10 min, 1 h, 2 h, 4 h, and 6 h by an IVIS Spectrum (PerkinElmer, MA, USA) with an excitation wavelength of 640 nm and an emission wavelength of 670 nm. The region of interest (ROI) was manually sketched to calculate the intensity of the fluorescence as mean fluorescence intensity (MFI). In addition, the tumors and main organs (bladder, liver, heart, lungs, stomach, spleen, skin, kidneys, colon, muscles, bones, and blood) were dissected and imaged for biodistribution analysis.

### 4.8. Data Analysis and Statistics

Statistical analysis was performed using GraphPad Prism software (v9.0, GraphPad, CA, USA). All data were presented as mean ± standard deviation (SD). The statistical significance between the groups was determined with either the two-tailed Student’s *t*-test or the one-way analysis of variance (ANOVA). The statistical significance threshold was set as *p* < 0.05 (* *p* < 0.05, ** *p* < 0.01, *** *p* < 0.001).

## 5. Conclusions

To sum up, an IGF2BP2-targeted peptide was successfully identified. In vitro and in vivo experiments confirmed that P12-Cy5 showed high affinity, specificity, and tumor-targeting properties. P12-Cy5 was found to be a prospective NIR probe for the imaging of ESCC.

## Figures and Tables

**Table 1:** The basic characteristics of ESCC patients.

Characteristics	Classification	Cases	Mean h-Score	SD of h-Score	*p* Value
Age (year)	≥60	60	191.5	61.9	0.445
	<60	38	200.5	45.9	
Gender	Male	71	196.6	57.1	0.648
	Female	27	190.7	54.6	
Lymph nodes	Positive	46	189.5	63.3	0.368
	Negative	52	199.8	49.1	
TNM stage	I	12	171.6	60.1	0.500
	II	23	197.7	67.5	
	III	58	198.2	50.1	
	IV	5	201.9	63.1	
Differentiation	Well	4	156.0	93.8	0.228
	Moderate	41	203.1	54.7	
	Poor	53	191.6	53.9	

## Data Availability

Data is contained within the article or supplementary material.

## Supplementary Materials

The following supporting information can be downloaded at: https://www.mdpi.com/article/10.3390/molecules27217609/s1, Figure S1: *IGF2BP2* expression profile in tumor tissues and normal tissues; Figure S2: The characteristic of P12 peptide.

## Sample Availability

Samples of the compounds are available from the authors.
