# Supplementary material for: Identification of an IGF2BP2-Targeted Peptide for Near-Infrared Imaging of Esophageal Squamous Cell Carcinoma
Source: Molecules. 2022 Nov 6;27(21):7609. doi: 10.3390/molecules27217609 (PMC9654546; doi:10.3390/molecules27217609)
Supplement: Supplementary file 1 [file molecules-27-07609-s001.zip › molecules-1991184-supplementary.pdf]

molecules

MDPI

Article

Identification of an IGF2BP2-Targeted Peptide for Near-Infrared Imaging of Esophageal Squamous Cell Carcinoma

Wenbin Shu [1,†], Yitai Xiao [2,†], Lizhu Wang [3,†], Mingzhu Liang [3], Zhihong Li [4], Xiangwen Wu [1,*] and Qingdong Cao [1,*]

[1] Department of Cardiothoracic Surgery, The Fifth Affiliated Hospital of Sun Yat-Sen University, Zhuhai 519000, China
[2] Guangdong Provincial Key Laboratory of Biomedical Imaging and Guangdong Provincial Engineering Research Center of Molecular Imaging, The Fifth Affiliated Hospital of Sun Yat-Sen University, Zhuhai 519000, China
[3] Department of Radiology, The Fifth Affiliated Hospital of Sun Yat-Sen University, Zhuhai 519000, China
[4] School of Medicine, South China University of Technology, Guangzhou 510006, China
* Correspondence: wuxw29@mail.sysu.edu.cn (X.W.); caoqd@mail.sysu.edu.cn (Q.C.)
† These authors contributed equally to this work.

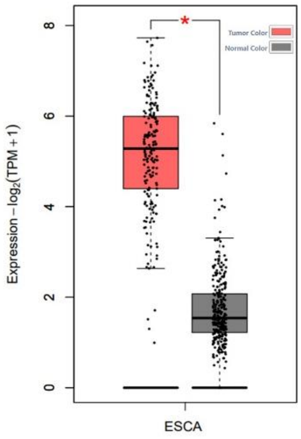

Figure S1. *IGF2BP2* expression profile in tumor tissues and normal tissues (TPM: transcript per million).

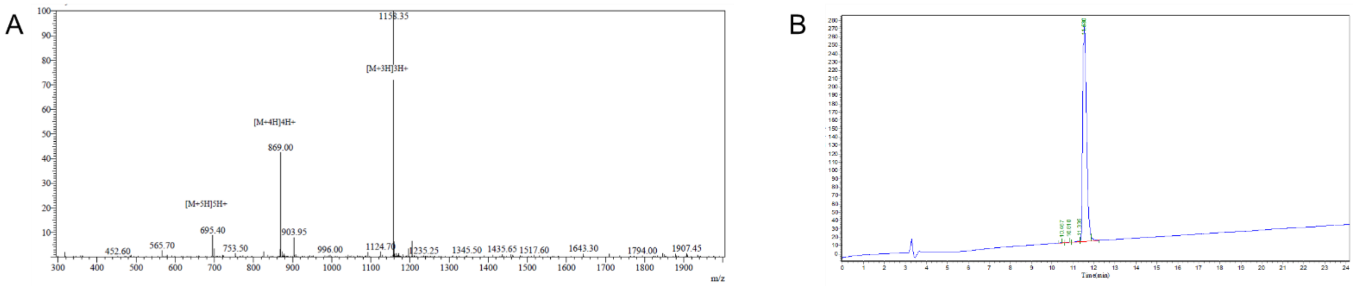

Figure S2. The characteristic of P12 peptide. (A) Mass spectrometry profile of P12 peptide. (B) The stability of P12 peptide analyzed by analytical HPLC.
